# Association of AI-Derived Quantitative CT Parameters of Airway, Emphysema, and Pulmonary Vasculature with Lung Cancer: A Cross-Sectional Analysis

**DOI:** 10.2174/0115734056413168251203110120

**Published:** 2026-01-15

**Authors:** Xiaojun Zhou, Qi Dai, Wei Lu, Zizhen Yang, Jianjun Zheng, Jingfeng Zhang

**Affiliations:** 1 Department of Radiology, Ningbo No.2 Hospital, Ningbo 315012, China

**Keywords:** Lung cancer, Computed tomography, Emphysema, Pulmonary vasculature, Airways, Chronic obstructive pulmonary disease

## Abstract

**Introduction:**

Given multiple risk factors for lung cancer, this study explored associations between lung cancer and AI-derived quantitative chest computed tomography (CT) parameters of emphysema, airways, and pulmonary vasculature.

**Methods:**

This retrospective single-center study (December 2020-February 2023) analyzed relevant parameters of the left upper lobe (LUL) and right upper lobe (RUL) in 170 lung cancer patients and 126 healthy individuals. Subgroups were defined by cancer-free lobes (129 patients/126 controls for LUL; 120 patients/126 controls for RUL). Univariate and multivariate binary logistic regression analyses were used for analysis.

**Results:**

The emphysema-related 15th percentile of CT attenuation values (PI-15) was significantly associated with lung cancer, with lower values in patients’ LUL. Pulmonary vascular parameters (diameter, count, area at 6 mm/24 mm from the lung surface) differed significantly; the patients had smaller diameters, higher counts, and larger areas at 6 mm in the LUL. Airway parameters (Awt-Pi10, level 6 wall thickness) were higher in patients’ LUL. Multivariate regression identified PI-15 and vascular diameters (6 mm/24 mm) in LUL [area under the curve (AUC) = 0.841, 95% confidence interval (95% CI): 0.789–0.892] and vascular diameters (6 mm/24 mm) and vascular count at 24 mm from the lung surface in RUL (AUC=0.819, 95% CI:0.766–0.872) as significant predictors (all *P*<0.001).

**Conclusion:**

AI-derived quantitative CT parameters of emphysema, vasculature, and airways are associated with lung cancer and may serve as complementary tools for clinical risk assessment.

## 1 INTRODUCTION

Although low-dose computed tomography (LDCT) screening in high-risk populations (individuals aged ≥ 40 years with a significant smoking history) has been shown to reduce lung cancer mortality [1], lung cancer remains the leading cause of cancer-related deaths worldwide [2-4]. This high mortality is partially attributed to the lack of specific early clinical manifestations and the challenges in accurately identifying high-risk individuals. While numerous risk factors, includingindoorairpollution,cigarettesmoking,cardio-vascular diseases, and chronic obstructive pulmonary disease (COPD), have been implicated in lung carcinogenesis [5-7], the development of interpretable quantitative biomarkers for risk stratification remains a critical unmet need. Over the past decade, alongside remarkable advances in therapeutic options for lung cancer, there has been a sustained effort to identify such biomarkers to enable effective risk stratification. Although multiple technologies (*e.g*., metabolomic profiling of exhaled breath and biofluids, which shows promising potential for the early detection and risk stratification of lung cancer) have been developed, their clinical translation remains pending [8-10].

Smoking, the leading risk factor for lung cancer, induces chronic airway inflammation, which in turn triggers airway wall thickening and emphysema, ultimately leading to irreversible destruction of lung structure. Quantitative CT cohort studies have shown that the presence of either emphysema or increased airway wall thickness elevates the risk of lung cancer mortality in smokers [11], whereas patients with COPD, particularly those with concurrent emphysema, exhibit a nearly threefold higher risk of lung cancer [12]. Emerging evidence indicates that integrating COPD detection into LDCT screening, especially *via* emphysema quantification, can improve lung cancer risk assessment [13, 14]. However, LDCT cannot quantify subtle abnormalities in the airways, pulmonary vasculature, or those related to emphysema (*e.g*., mild airway wall thickening or reduced vascular branching) that precede lung nodule formation, resulting in delayed risk identification.

With rapid advancements in computational technologies, AI can extract objective, high-dimensional parameters that are unmeasurable *via* visual LDCT assessment and has been increasingly used to characterize pulmonary nodules, analyze the aggressive biological phenotypes of lung cancer, and predict patient outcomes [15-17]. Recent progress in CT image segmentation techniques now enables the quantification of pulmonary vasculature, emphysema, and airways. For instance, CT-derived parameters for airway dimensions include airway lumen area (Ai) and airway wall area percentage (WA%, defined as the ratio of airway wall area to the sum of wall and lumen areas), while emphysema is quantitatively assessed using image voxels with a density of < -950 Hounsfield Units (HU) [18-21]. Most existing radiomics studies on lung cancer focus on tumor-related features [22]. In contrast, our study integrated three interdependent pulmonary components, airway, emphysema, and pulmonary vasculature, to capture comprehensive lung structural abnormalities associated with lung cancer. Previous AI models for lung cancer risk assessment often rely on subjective image features or single-modal data [23]. Our model, by contrast, uses fully automated AI algorithms to extract quantitative parameters (with no manual intervention) and links multi-structural features to cancer risk. This approach addresses the “black box” problem of some AI models by linking parameters to interpretable biological relevance.

This study aimed to investigate correlations between CT-based quantitative parameters of emphysema, pulmonary airways, and vasculature in lung cancer patients and those of healthy controls, with the goal of identifying interpretable high-risk biomarkers to enhance LDCT screening strategies.

## 2 METHODS

### 2.1 Study Population

**Fig. (1):**
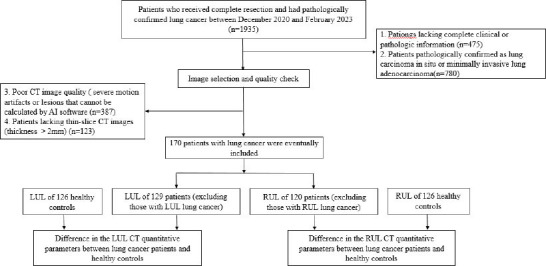
Flowchart of study design and patient inclusion/exclusion pathway. LUL: left upper lobe; RUL: right upper lobe.

A total of 296 participants, comprising 126 healthy individuals and 170 lung cancer patients from Ningbo No. 2 Hospital, all of whom underwent a CT scan between December 2020 and February 2023, were enrolled in this retrospective study. The study was approved by the ethics committee of Ningbo No. 2 Hospital (grant no.: 2022-112-1). The exclusion criteria were as follows: (a) participants lacking complete clinical or pathological information; (b) pathological diagnosis of carcinoma *in situ* or minimally invasive lung cancer (applicable to lung cancer patients); (c) participants lacking continuous thin-slice CT images; and (d) insufficient CT image quality (*e.g*., severe motion artifacts or lesions that could not be evaluated by AI software). Ultimately, 129 patients (selected from the 170 lung cancer patients, with no lung cancer in LUL) were allocated to the LUL group, and 120 patients (also selected from the 170 lung cancer patients, with no lung cancer in RUL) were allocated to the RUL group. The 126 healthy individuals served as controls for both groups (Fig. **1**).

### 2.2 Chest CT Scan

Chest CT was performed on Siemens Somatom Force, Siemens Definition AS, UIH uCT710, and GE Optima CT540 scanners. The scanning coverage extended from the lung apex to the costophrenic angles, including bilateral chest walls and axillary regions. Scanning parameters for all four devices were set as follows: tube voltage of 70–100 kV and tube current of 300–400 mA (automatically adjusted according to patient habitus). Image analysis was performed using lung window settings (window width: 1500 HU, window level: -400 HU).

### 2.3 Quantitative Post-processing

**Fig. (2):**
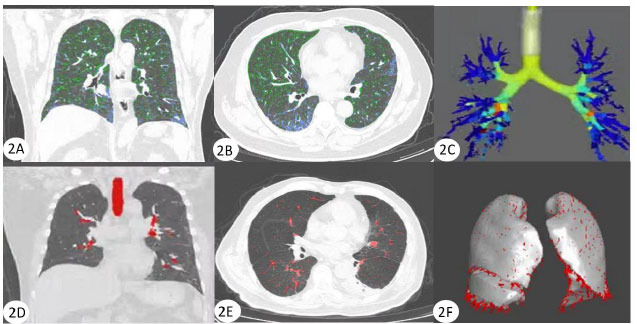
Application of automated AI algorithms for quantifying pulmonary structures from CT scans. Subpanels are illustrated as follows: (**A** and **B**) show emphysema extent mapping (with a density mask threshold of -950 HU); (**C** and **D**) show airway tree reconstruction; and (**E** and **F**) show pulmonary vasculature segmentation.

The commercial AI-based AVIEW COPD platform (Coreline, Seoul, Korea; version 1.1.46.11-win) automatically analyzed chest CT images (Fig. **2A-F**). This software enables automated whole-lung parameter analysis, followed by the extraction of quantitative metrics from the LULs and RULs.

Emphysema-related parameters derived from CT scans included low attenuation area volume (LAA_Volume, cm^3^), low attenuation area volume percentage (LAA_Volume%), mean Hounsfield Units (mean, HU), standard deviation of HU (Std, HU), and skewness/kurtosis of lung lobe density distributions. The PI-15 was additionally incorporated as a key emphysema parameter. For binary classification of emphysema, CT values ≤ -950 HU were defined as 1, and values > -950 HU were defined as 0.

Pulmonary vasculature parameters were measured at two distances from the lung surface: 6 mm and 24 mm. At 6 mm, parameters included mean vascular diameter (DiameterMean_ 6mm, mm), total vessel area (VesselArea_6mm, mm^2^), and vascular counts (NumVessels_6mm, units). The corresponding 24 mm parameters comprised mean vascular diameter (DiameterMean_ 24mm, mm), total vessel area (VesselArea_24mm, mm^2^), and vascular counts (NumVessels_24mm, units).

Airway parameters, as depicted in Fig. (**3**), included wall thickness (WT), airway wall area percentage (WA%, relative to the sum of wall and lumen areas), airway lumen area (Ai), and the 10th percentile of airway wall thickness normalized by the square root of WA% for airways with an internal perimeter of 10 mm (Awt-Pi10). CT-derived airway metrics encompassed whole-lung Awt-Pi10 (Awt-Pi10_whole, mm), Awt-Pi10 for level 5 airways (Awt-Pi10_Level5, mm), WT of level 5 airways (WT_Level5, mm), WA% of level 5 airways (WA%_Level5, %), Ai of level 5 airways (Ai_Level5, mm^2^), and the corresponding parameters for level 6 airways (Awt-Pi10_Level6, mm; WT_Level6, mm; WA%_Level6, %; Ai_Level6, mm^2^).

### 2.4 Statistical Analysis

All statistical analyses were conducted using SPSS Statistics (Version 23.0; IBM, Armonk, NY, USA) and R (Version 4.3.3; R Foundation for Statistical Computing, Vienna, Austria). Baseline characteristics were compared using the Student’s *t*-test and Mann-Whitney U test for continuous variables, and the chi-square test for categorical variables. Univariate binary logistic regression analysis of AI-derived CT quantitative parameters (emphysema, pulmonary vasculature, and airways) in LUL/RUL was performed, with results presented as odds ratios (OR) and 95% CI. Variables significant in univariate analysis (*P* ≤ 0.05) were included in Lasso (least absolute shrinkage and selection operator) regression to address multicollinearity and variable selection. Eventually, the selected variables were included in multivariable logistic regression for each lobe (LUL and RUL), and the variance inflation factor (VIF) of the final model was calculated to assess collinearity. Model accuracy was evaluated using receiver operating characteristic (ROC) curves, and the area under the ROC curve (AUC) was calculated. The results of multivariate binary logistic regression were visualized using a nomogram.

## 3 RESULTS

### 3.1 Participant Characteristics

Overall, 170 lung cancer patients and 126 healthy controls were included in this analysis (Fig. **1**). The mean age of the participants was 65.3±10.2 years (64.3±9.9 years in patients *vs*. 66.7±10.4 years in controls). Regarding demographic and clinical characteristics, 157 participants (53%) were male (77 in the patient group *vs*. 80 in the control group), and 71 (24%) had a history of smoking (37 in the patient group *vs*. 34 in the control group). Approximately 49 cases exhibited CT findings indicative of chronic bronchitis (40 in the patient group *vs*. 9 in the control group).

### 3.2 Difference of Emphysema CT Quantitative Parameters between Lung Cancer and Healthy Controls

The emphysema index PI-15 in LUL was significantly associated with lung cancer. Specifically, the proportion of participants with LUL CT values ≤ -950 HU was lower in the patient group than in the control group (13/116 *vs*. 41/85; OR = 0.230, *P* = 0.001). Other quantitative emphysema parameters in the LUL exhibited no statistically significant differences, as shown in Table **1**.

### 3.3 Pulmonary Vasculature Analysis between Lung Cancer Patients and Healthy Controls

Baseline differences in pulmonary vascular parameters between lung cancer patients and controls are detailed in Table **2**.

At 6 mm from the lung surface, vascular diameters were significantly smaller in patients than in controls for both lobes (LUL: 1.94 ± 0.20 mm *vs*. 2.30 ± 0.50 mm; OR = 0.036, 95% CI: 0.014–0.096, *P* = 0.001; RUL: 1.94 ± 0.17 mm *vs*. 2.21 ± 0.40 mm; OR = 0.049, 95% CI: 0.018–0.133, *P* = 0.001). At 24 mm from the lung surface, comparable reductions in vascular diameters were observed in both lobes (LUL: 2.90 ± 0.27 mm *vs*. 3.32 ± 0.48 mm; OR = 0.077, 95% CI: 0.036–0.167, *P* = 0.001; RUL: 2.90 ± 0.28 mm *vs*. 3.30 ± 0.49 mm; OR = 0.085, 95% CI: 0.040–0.183, *P* = 0.001).

Pulmonary vessel area exhibited distance-dependent differences between the two groups; specifically, total vessel areas were larger in patients than in controls at 6 mm from the lung surface in both lobes (LUL: 1091.1 ± 359.4 mm^2^ *vs*. 911.5 ± 531.8 mm^2^; OR = 1.001, 95% CI: 1.000–1.001, *P* = 0.002; RUL: 848.0 ± 279.6 mm^2^ *vs*. 689.6 ± 479.7 mm^2^; OR = 1.001, 95% CI: 1.000–1.002, *P* = 0.003), yet smaller in patients at 24 mm (LUL: 393.0 ± 256.5 mm^2^ *vs*. 477.2 ± 286.7 mm^2^; OR = 0.999, 95% CI: 0.988–1.000, *P* = 0.016; RUL: 310.4 ± 167.2 mm^2^ *vs*. 462.7 ± 238.3 mm^2^; OR = 0.996, 95% CI: 0.995–0.998, *P* = 0.001).

Pulmonary vascular counts also differed significantly between the groups. At 6 mm from the lung surface, patients had more vessels in both lobes than in controls (LUL: 358.4 ± 110.7 *vs*. 227.4 ± 169.8; OR = 1.006, 95% CI: 1.004–1.008, *P* = 0.001; RUL: 301.8 ± 80.0 *vs*. 207.0 ± 159.0; OR = 1.006, 95% CI: 1.004–1.008, *P* = 0.001), while at 24 mm, fewer vessels were observed in patients than in controls in the RULs (46.6 ± 20.9 *vs*. 57.0 ± 24.1; OR = 0.979, 95% CI: 0.968–0.991, *P* = 0.001).

### 3.4 Airway Quantitative Parameters Analysis between Lung Cancer Patients and Healthy Controls

As shown in Table **3**, the patients exhibited statistically significant differences in the following two airway parameters compared with the controls: the Awt-Pi10_Whole (4.24 ± 1.00 mm *vs*. 4.00 ± 0.79 mm; OR = 1.407, 95% CI: 1.059–1.871, *P* = 0.018) and WT_Level6 in the LUL (1.78 ± 0.71 mm *vs*. 1.61 ± 0.54 mm; OR = 1.558, 95% CI: 1.035–2.345, *P* = 0.033).

### 3.5 Lasso and Multivariate Logistic Regression Analysis of Emphysema, Pulmonary Vessels, and Airways in Bilateral Upper Lobes

**Fig. (4):**
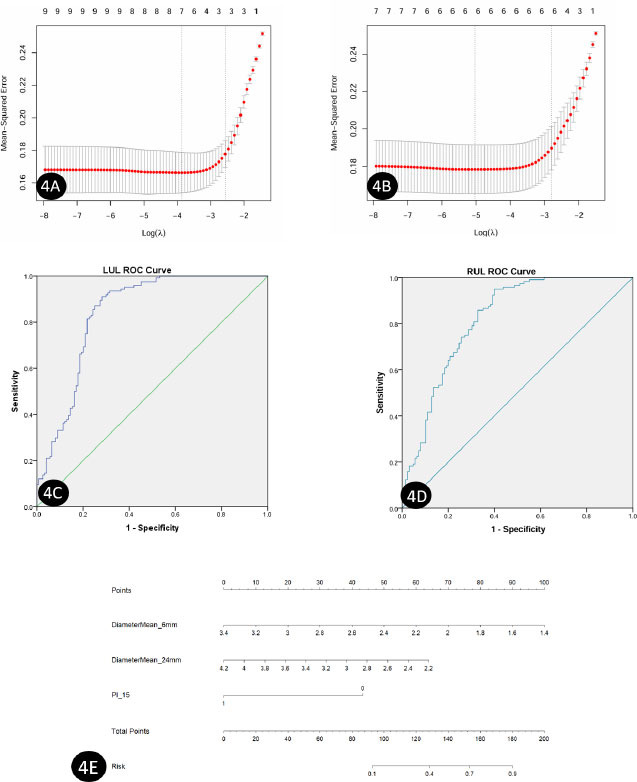
LASSO coefficient profiles and ROC curves for evaluating the discriminative performance of multivariable logistic models in lung cancer. Panels **4A** (LUL) and **4B** (RUL) depict the standardized regression coefficients of each imaging feature as a function of log(λ). Panel **4C** shows the ROC curve of the LUL model (AUC = 0.841; 95% CI: 0.789–0.892; *P* < 0.001); panel **4D** shows the ROC curve of the RUL model (AUC = 0.819; 95% CI: 0.766–0.872; *P* < 0.001); and panel **4E** presents the nomogram derived from the LUL model.

First, we separately subjected the indicators with significant univariate results for the LUL and RUL to Lasso regression analysis, aiming to address multicollinearity and select optimal variables for further analysis (Fig. **4A and B**). Subsequently, the results of the multivariate logistic regression analysis are presented in Table **4**.

For the LULs, three variables were ultimately retained: the PI-15, mean vascular diameter at 6 mm from the lung surface (mm), and mean vascular diameter at 24 mm from the lung surface (mm). Subsequent multivariate logistic regression analysis confirmed these three variables as significant predictors of lung cancer, with the model yielding an AUC of 0.841 (95% CI: 0.789–0.892, *P* < 0.001; (Fig. **4C**). We further assessed multicollinearity among these variables, and the corresponding VIF values were 1.15, 1.55, and 1.52, matching PI-15, vascular diameter at 6 mm, and vascular diameter at 24 mm, respectively.

For the RUL, the vascular area at 6 mm from the lung surface (mm^2^) was excluded after Lasso regression. Among the remaining variables, vascular area at 24 mm from the lung surface (mm^2^) and chronic bronchitis were further eliminated following incorporation into multivariate logistic regression. The final regression model for RUL identified three significant predictors of lung cancer for further analysis: (*i.e*., vascular diameters at 6 mm and 24 mm from the lung surface, along with vascular count at 24 mm from the lung surface), with an AUC of 0.819 (95% CI: 0.766-0.872, *P* < 0.001; Fig. **4D**). The VIF values of variables in the final RUL model ranged from 1.69 to 1.72, indicating no significant multicollinearity. The multivariate regression results for the LUL are visualized in Fig. (**4E**).

## 4 DISCUSSION

In the present study, we detected distinct intergroup differences in emphysema, pulmonary airway, and pulmonary vascular parameters in the bilateral upper lobes when comparing lung cancer patients with healthy controls. Compared with conventional radiomics models, the quantitative variables included in our study exhibited explicit interpretability and stronger clinical generalizability. It is widely recognized that parameters related to emphysema, pulmonary airways, and pulmonary vasculature undergo substantial alterations in individuals with COPD and current or former smokers. However, the mechanistic links between these quantitative indices and lung cancer pathogenesis remain incompletely understood.

The results of this study demonstrated significant differences in vascular architecture at two distinct lung depths: 6 mm from the lung surface (peripheral compartment) and 24 mm from the lung surface (central compartment). In the central compartment, vascular diameter and vascular density were lower in controls, whereas in the peripheral compartment, vascular diameter was lower, yet vascular density was higher in controls. This biphasic remodeling pattern suggested potential peripheral angiogenesis and central obstructive remodeling. Smokers and patients with COPD often exhibit varying degrees of pulmonary vascular remodeling, characterized by a reduced number and size of small pulmonary vessels [24-26], a pattern that differs from our findings. This discrepancy may stem from the following two potential mechanisms: first, in lung cancer patients, the increase in peripheral small blood vessels may be associated with the abnormal tumor microenvironment. High-resolution CT parameters reflect the spatial heterogeneity of the tumor microenvironment; these cancer neovessels exhibit increased basal lamina thickness and irregular caliber, which translate into larger mean diameters on CT and greater intratumoral perfusion heterogeneity [27-29]. However, the precise mechanisms underlying the systematic alteration of the peripheral vasculature in lung cancer patients remain to be further elucidated. Second, computational artifacts from AI segmentation algorithms may fail to account for fibrotic bands in CT images. It is worth mentioning that these results highlight both potential shared and unique vascular remodeling patterns between COPD and lung cancer, emphasizing the need for further mechanistic exploration.

Pulmonary emphysema has emerged as an established risk factor for lung carcinogenesis [30, 31]. Previous studies have demonstrated that LAA and PI-15 can serve as independent predictors of smoking-related lung injury [32, 33]. In the present study, PI-15 in LUL exhibited a statistically significant association with lung cancer (OR = 10.416, 95% CI: 4.091–26.519, *P* ≤ 0.001). Since PI-15 reflects the 15th percentile of lung tissue CT attenuation values, this result implies that lung cancer patients have lower lung attenuation values in LUL, consistent with potential emphysema-related density reductions. Notably, as one of the established high-risk factors for lung cancer, this study found that the incidence of chronic bronchitis was higher in the enrolled lung cancer cases than in the general population.

Airway remodeling, particularly involving small airways (<2 mm in diameter), represents a cardinal pathological feature of COPD [34]. Previous tomographic studies have demonstrated preferential involvement of the upper lobes in COPD-related airway remodeling [35, 36]. Our study showed that both Awt-Pi10 and the WT of level 6 airways in LUL were greater in lung cancer patients than in controls (*P* < 0.05), consistent with previous observations, potentially indicating the presence of pulmonary airway remodeling. However, no significant differences in these airway parameters were observed in the RUL. The specific pathophysiological mechanisms underlying this lobe-specific discrepancy warrant further exploration.

### 4.1 The Value of Clinical Application

This study revealed that the selected parameters of emphysema, pulmonary vasculature, and airways in LUL showed statistically significant differences between lung cancer patients and healthy controls, whereas only pulmonary vascular parameters in the RUL demonstrated such significance. Intriguingly, after adjusting for clinically relevant covariates (*e.g*., age and smoking history), our analysis confirmed significant differences in LUL PI-15 among emphysema indices and pulmonary vascular parameters. In contrast, statistical significance in the RUL was limited to pulmonary vascular parameters alone. Notably, the magnitude of these differences was more pronounced in the LUL, specifically for the predictive performance of the models, evidenced by the AUC values of 0.841 for the LUL model (95% CI: 0.789–0.892, *P* < 0.001) and 0.819 for the RUL model (95% CI: 0.766–0.872, *P* < 0.001). These findings underscore the importance of a comprehensive assessment of pulmonary vasculature.

### 4.2 The Limitations of Study

The limitations of our study should be noted. First, this was a single-center retrospective study, leading to a relatively small sample size. To mitigate this and enhance result reliability, we separately analyzed the quantitative parameters of LUL and RUL by comparing them with healthy controls. However, multicenter studies with larger sample sizes are still needed to validate the effectiveness and the practicality of the aforementioned model. Moreover, we plan to enroll additional participants in a prospective study to further verify our findings. Second, to maximize detectable between-group differences, this study exclusively enrolled patients with solid-density lung cancer, thus requiring further research to explore whether similar differences exist in patients with earlier-stage lung cancer, specifically those presenting with part-solid density or ground-glass density on CT. Third, the limited discriminative ability of our model can be partially attributed to insufficient data on patients with comorbid COPD and lung cancer. In future studies, we will expand our cohort to include larger, well-characterized groups of patients with comorbid COPD and lung cancer to address this limitation.

## CONCLUSION

Quantitative CT parameters of the airways, emphysema, and pulmonary vasculature derived from artificial intelligence (AI) are closely linked to lung cancer. The results of this study confirm that these AI-based metrics show promise as supplementary indicators to support the evaluation of lung cancer risk in clinical settings.

## Figures and Tables

**Fig. (3):**
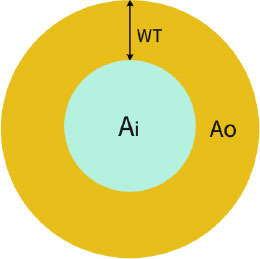
Quantitative CT parameters of airway wall. Ai: Airway lumen area AO: Total airway area WA% = (Ai/AO) × 100% WT: Airway wall thickness

**Table 1:** Univariate analysis of emphysema in bilateral upper lobes between lung cancer patients and healthy controls.

Variables	Left upper lobe				Right upper lobe			
	Lung cancer (n=129)	Controls (n=126)	*P1*	OR (95% CI)	Lung cancer (n=120)	Controls (n=126)	*P2*	OR (95% CI)
Smoking	27 (21%)	34 (27%)	0.243	/	25 (21%)	34 (27%)	0.260	/
Chronic bronchitis	26 (20%)	9 (7%)	0.004	3.285 (1.471~7.336)	26 (22%)	9 (7%)	0.002	3.596 (1.607-8.044)
LAA_Volume (cm^3^)	51.2±85.5	37.2±71.2	0.569	/	30.0±58.1	24.2±53.7	0.431	/
LAA_Volume (%)	4.8±6.6	3.7±6.9	0.189	/	3.9±6.6	3.1±6.6	0.375	/
Mean (HU)	-816.0±49.1	-810.9±42.9	0.380	/	-815.9±45.4	-812.6±38.2	0.534	/
Std (HU)	142.4±19.7	142.2±19.6	0.934	/	138.7±18.5	135.1±24.1	0.200	/
Skewness	3.2±0.6	3.2±0.5	0.727	/	3.3±0.5	3.3±0.5	0.271	/
Kurtosis	16.5±4.7	16.4±4.5	0.883	/	16.8±4.4	17.1±4.0	0.482	/
PI-15 (1/0)	13/116	41/85	0.001*	0.230 (0.116-0.455)	10/110	7/119	0.394	/

**Note:** Data are presented as mean ± SD. The asterisk (*) denotes statistically significant differences. PI-15: CT values ≤ -950 HU were defined as 1, and values > -950 HU were defined as 0.

**Table 2:** Univariate analysis of lung vasculature parameters in bilateral upper lobes between lung cancer patients and healthy controls.

**Variables**	**Left upper lobe**						**Right upper lobe**	
	**Lung cancer (n=129)**	**Controls (n=126)**	***P1***	**OR (95% CI)**	**Lung cancer (n=120)**	**Controls (n=126)**	***P2***	**OR (95% CI)**
DiameterMean(mm) _6mm	1.94±0.20	2.30±0.50	0.001*	0.036 (0.014-0.096)	1.94±0.17	2.21±0.40	0.001*	0.049 (0.018-0.133)
VesselArea (mm^2^)_6mm	1091.1±359.4	911.5±531.8	0.002*	1.001 (1.000-1.001)	848.0±279.6	689.6±479.7	0.003*	1.001 (1.000-1.002)
NumVessels(units)_6mm	358.4±110.7	227.4±169.8	0.001*	1.006(1.004-1.008)	301.8±80.0	207.0±159.0	0.001*	1.006(1.004-1.008)
DiameterMean(mm)_24mm	2.90±0.27	3.32±0.48	0.001*	0.077 (0.036-0.167)	2.90±0.28	3.30±0.49	0.001*	0.085 (0.040-0.183)
VesselArea (mm^2^_24mm	393.0±256.5	477.2±286.7	0.016*	0.999 (0.988-1.000)	310.4±167.2	462.7±238.3	0.001*	0.996 (0.995-0.998)
NumVessels (units)_24mm	67.8±42.4	61.7±31.2	0.191	/	46.6±20.9	57.0±24.1	0.001*	0.979 (0.968-0.991)

**Note:** Data are presented as mean ± SD. The asterisk (*) denotes statistically significant differences.

**Table 3:** Univariate analysis of pulmonary airways in bilateral upper lobes between lung cancer patients and healthy controls.

Airways variables	Left upper lobe				Right upper lobe			
-	Lung cancer (n=129)	Controls (n=126)	*P1*	OR (95% CI)	Lung cancer(n=120)	Controls (n=126)	*P2*	OR (95% CI)
Awt-Pi10 (mm)_Whole	4.24±1.00	4.00±0.79	0.018*	1.407(1.059-1.871)	3.95±0.97	3.88±0.86	0.560	/
Awt-Pi10 (mm)_Level5	7.82±23.7	6.25±6.03	0.530	/	4.59±2.47	3.81±7.23	0.389	/
WT (mm)_Level5	2.29±0.69	2.37±0.78	0.384	/	1.94±0.88	1.89±0.65	0.606	/
WA%_Level5	73.0±8.60	73.5±8.02	0.664	/	70.6±10.5	69.6±11.3	0.482	/
Ai (mm^2^)_Level5	19.7±18.14	18.9±11.44	0.681	/	14.4±9.8	16.8±17.0	0.237	/
Awt-Pi10 (mm)_Level6	5.00±4.30	4.71±1.65	0.494	/	4.03±1.96	4.07±2.37	0.886	/
WT (mm)_Level6	1.78±0.71	1.61±0.54	0.033*	1.558(1.035-2.345)	1.30±0.58	1.33±0.62	0.632	/
WA %_Level6	70.2±10.4	68.1±9.5	0.095	/	65.6±10.8	64.4±10.3	0.409	/
Ai (mm^2^)_Level6	12.1±8.6	11.5±6.8	0.511	/	9.5±6.0	9.6±7.0	0.862	/

**Note:** Data are presented as mean ± SD. The asterisk (*) denotes statistically significant differences.

**Table 4:** Multivariate analysis of pulmonary airways in bilateral upper lobes between lung cancer patients and healthy controls.

-	LUL (n=255)		RUL (n=246)	
	OR (95% CI)	*P*-value	OR (95% CI)	*P*-value
Chronic bronchitis	/	/	/	/
PI-15 (HU)	10.416 (4.091~26.519)	0.001*	/	/
DiameterMean(mm) _6mm	0.023 (0.004~0.147))	0.001*	0.127 (0.023~0.713)	0.019*
VesselArea(mm^2^)_6mm	/	/	/	/
NumVessels(ea)_6mm	/	/	/	/
DiameterMean(mm)_24mm	0.132 (0.028~0.630)	0.008*	0.170 (0.047~0.618)	0.007*
VesselArea(mm^2^)_24mm	/	/	/	/
NumVessels(units)_24mm	/	/	0.956 (0.926~0.988)	0.007*
BAND_Awt-Pi10(mm)_Whole	/	/	/	/
BAND_WTMean(mm)_Level6	/	/	/	/

**Note:** Data are presented as mean ± SD. The asterisk (*) denotes statistically significant differences.

## Data Availability

The data supporting this study's findings will be available upon reasonable request from the corresponding author [J. Z.].

## LIST OF ABBREVIATIONS

CT: Computed tomography
LDCT: Low-dose computed tomography
LUL: Left upper lobe
RUL: Right upper lobe
AI: Artificial intelligence
OR: Odds ratio
COPD: Chronic obstructive pulmonary disease
ROC: Receiver operating characteristic
AUC: Area under the curve
95% CI: 95% confidence interval
VIF: Variance inflation factor
LASSO: Least absolute shrinkage and selection operator
WT: Wall thickness

## STANDARDS OF REPORTING

STROBE guidelines were followed.
